# Supplementary material for: Causal Relationship between Aging and Anorexia Nervosa: A White-Matter-Microstructure-Mediated Mendelian Randomization Analysis
Source: Biomedicines. 2024 Aug 16;12(8):1874. doi: 10.3390/biomedicines12081874 (PMC11351342; doi:10.3390/biomedicines12081874)
Supplement: Supplementary file 1 [file biomedicines-12-01874-s001.zip › Supplementary Figure/Figure S8/Leaveoneout plot of MR analysis for TL to ubm-b-1874.pdf]

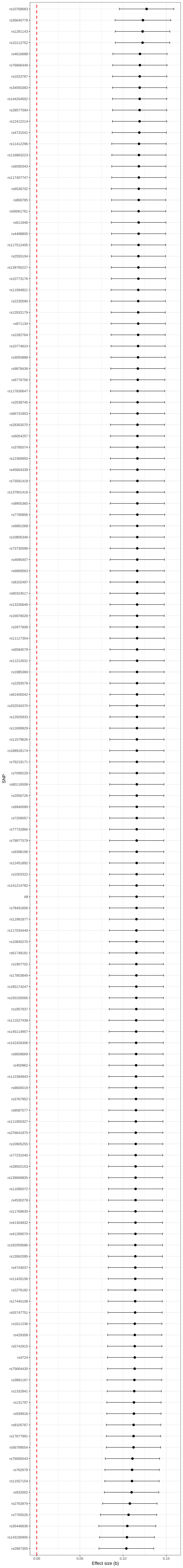

rs10768683
rs35640778
rs1291143
rs10112752
rs4616688
rs76666449
rs1023767
rs34550383
rs144204502
rs28577594
rs12412214
rs4731541
rs11412296
rs116863223
rs6590343
rs117407747
rs6536702
rs869785
rs56061761
rs611646
rs4498805
rs117512405
rs2555104
rs139795227
rs10773176
rs11584821
rs2230590
rs12932179
rs871134
rs2282764
rs10774624
rs3093888
rs9878436
rs6776756
rs117630647
rs2538745
rs66731853
rs28363070
rs6054257
rs3785074
rs12369950
rs45604339
rs73581419
rs137901416
rs9955360
rs7790856
rs6881568
rs10805346
rs73730598
rs4695407
rs6669563
rs8102497
rs80324517
rs13230646
rs16978028
rs2977608
rs11117354
rs6584579
rs11212631
rs1985369
rs2293579
rs61405042
rs202034370
rs12925933
rs11699829
rs11579626
rs188918174
rs76219171
rs7099229
rs80116508
rs2056726
rs9940099
rs7209057
rs77732866
rs79977579
rs9398196
rs12451892
rs1003322
rs141214782
All
rs78491606
rs11991877
rs117034449
rs10840270
rs61748181
rs1907702
rs17803849
rs185174247
rs150150565
rs1957937
rs111527438
rs145114957
rs142426306
rs6659669
rs450962
rs112394943
rs9600019
rs3767952
rs6587577
rs111950327
rs376641875
rs10905255
rs77231040
rs28502153
rs139669835
rs11085072
rs4530278
rs11769630
rs41304832
rs41269079
rs182059586
rs13062095
rs4743037
rs11426156
rs2276182
rs17445108
rs55747751
rs1611236
rs429358
rs5742915
rs4724
rs75664430
rs3891167
rs1332941
rs131797
rs939916
rs8105767
rs17677991
rs56799554
rs76065543
rs762679
rs11557154
rs932002
rs2763979
rs7705526
rs35446936
rs143190905
rs2967355
SNP
0.00
0.05
0.10
0.15
Effect size (b)
